# Ultrasound-assisted deep eutectic solvent extraction versus alkaline extraction: Functional and structural properties of hazelnut proteins

**DOI:** 10.1016/j.fochx.2025.103080

**Published:** 2025-09-24

**Authors:** Esra Kibar Balballi, Gulsah Karabulut

**Affiliations:** Department of Food Engineering, Faculty of Engineering, Sakarya University, Sakarya, 54187, Türkiye

**Keywords:** Hazelnut meal, Deep eutectic solvent, Protein extraction, Emulsifying activity, Digestibility

## Abstract

This study compared conventional alkaline extraction and ultrasound-assisted deep eutectic solvent (DES–US) extraction for isolating functional proteins from hazelnut meal, an underutilized oilseed by-product. Alkaline extraction at pH 10 achieved the highest protein recovery (260 mg/g) and extraction efficiency (63 %), while DES–US systems yielded moderate soluble recoveries (∼200–220 mg/g) but showed reduced final protein yields (80 mg/g) due to precipitation challenges. Molecular weight profiles confirmed that both methods effectively preserved major storage proteins, including 7S and 11S globulins and 2S albumins. Secondary structural analyses revealed pronounced pH-induced denaturation in alkaline extracts, versus high α-helix retention in DES–US samples (e.g., DES-GLU 95.7 %, DES-SOR 81.6 %). DES–US systems also exhibited higher solubility (75–76 %) compared to alkaline extracts (58–63 %). Emulsifying activity index was significantly higher for DES–US (DES-GLY 15 m^2^/g) than alkaline (<6 m^2^/g), while emulsion stability peaked at ∼60 min for DES-SOR versus ∼5–6 min for alkaline samples. Conversely, alkaline extracts demonstrated superior foaming capacity (∼50 % for ALK10) and stability (∼40–50 % at 120 min), while DES–US foams were less stable (∼10–25 % FC). In vitro digestibility was highest for DES–US isolates (DES-SOR ∼76 %, DES-GLY ∼75.1 %) compared to alkaline treatments (∼57.6–65.3 %). These results suggest DES–US extraction offers a greener, scalable strategy for producing hazelnut proteins with improved solubility, emulsifying properties, and digestibility, supporting sustainable ingredient development from agro-industrial residues.

## Highlights

• DES–US extraction preserved hazelnut protein structure better than alkaline extraction.
• DES–US isolates showed higher solubility, emulsifying activity, and digestibility.
• Alkaline extraction gave higher yields and foaming capacity.
• DES–US offers a greener strategy to produce functional plant proteins.

## Graphical abstract

**Figure:**
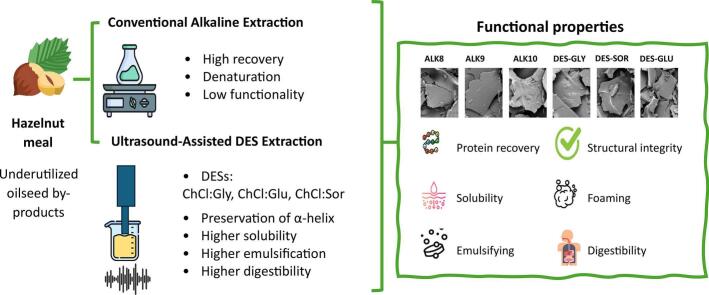

## 1 Introduction

The increasing urgency to transition toward sustainable food systems has intensified global interest in alternative protein sources that are both environmentally viable and functionally robust. By 2021, nearly 10 % of the world's population faced hunger, with Africa exhibiting the highest and fastest-growing rates of undernourishment (FAO, 2022). Addressing hunger requires improving access, affordability, and the resilience of food systems; at the same time, expanding protein supply must avoid reproducing the environmental inefficiencies of conventional animal agriculture. Against this background, animal-based protein production typically entails higher greenhouse gas emissions, more intensive water and land use, and ethical concerns, motivating a paradigm shift toward plant-based proteins and the valorization of agri-food waste streams (McClements & Grossmann, 2024). Such shifts are critical not only to meeting global climate goals and reducing environmental footprints but also to expanding equitable, sustainable protein access for a growing population.

Among underutilized plant-based resources, agro-industrial residues such as oilseed meals represent promising substrates for integration into the circular bioeconomy. Hazelnut meal, a defatted by-product of cold-press oil extraction, is particularly protein-rich (up to 54.4 % by dry weight) but remains largely underexploited for human consumption, often relegated to animal feed or discarded (Ceylan et al., 2023). Given Türkiye's status as the world's leading hazelnut producer, valorizing hazelnut meal aligns with regional bioeconomic goals while contributing to global sustainability targets. Hazelnut proteins also exhibit favorable techno-functional properties, including emulsification and foaming, making them attractive candidates for functional food formulations (Jiang et al., 2024).

However, successful incorporation into food systems requires extraction methods that retain native structure and functionality while minimizing environmental impact. Conventional alkaline extraction remains the dominant method for solubilizing plant proteins due to its simplicity and high yield. However, it often induces partial denaturation, reduces solubility, and compromises functional properties, particularly at high pH, where disulfide linkages are disrupted and aggregation is promoted (Anzà et al., 2025). In addition to impairing protein quality, alkaline extraction generates large volumes of chemical waste and requires significant water consumption, challenging its sustainability credentials (Cao et al., 2023; Hanafi et al., 2024). Alkaline treatments have also been shown to result in dark-colored, less appealing protein fractions due to phenolic oxidation and Maillard-type reactions (Đermanović et al., 2025; Karimi et al., 2024).

These limitations underscore the need for alternative extraction strategies that are both environmentally benign and function-preserving. In this context, deep eutectic solvents (DESs) have emerged as a promising class of green solvents for protein extraction. DESs are formed through hydrogen bonding between a hydrogen bond acceptor (HBA), such as choline chloride or betaine, and a donor (HBD) like urea, glycerol, or organic acids (Marinaccio et al., 2024; Marinaccio et al., 2025). They are characterized by low volatility, biodegradability, and strong solubilization potential for biomacromolecules (Hanafi et al., 2024; Olalere & Gan, 2023). Particularly, the subclass of natural deep eutectic solvents (NADES), composed entirely of food-grade metabolites, is especially suited for food applications due to low cytotoxicity and safe status (Guzmán-Lorite et al., 2022; Lores et al., 2017). DES systems have been extensively studied for a range of plant matrices, including oilseed meals (Cao et al., 2023; Đermanović et al., 2025), nut by-products (Jiang et al., 2024), and leaf proteins (Patra & Venugopal, 2025), demonstrating their ability to extract proteins with higher purity, improved solubility, and better retention of essential amino acids compared to traditional solvents.

To further improve extraction efficiency and minimize protein damage, ultrasound (US)-assisted extraction has been increasingly applied in combination with DES systems. The mechanical effects of acoustic cavitation, including microjet formation, cell wall rupture, and improved solvent penetration, accelerate mass transfer and enhance protein release under mild thermal conditions (Gao et al., 2022). US-DES combinations have shown synergistic effects in fava beans (Hewage et al., 2024), brewer's spent grain (Hadinoto et al., 2025), and mushroom by-products (Karabulut et al., 2024), yielding improved protein recovery, functional retention, and structural integrity compared to conventional solvents.

Despite these promising results in various plant systems, hazelnut meal remains largely unexplored as a substrate for DES-based or US-assisted DES extractions. The influence of DES formulation (e.g., HBD type, molar ratio, water content) on hazelnut protein yield, purity, and structural properties is not well established, nor has it been systematically compared to alkaline extraction in terms of functional behavior such as solubility, emulsification, and foaming. Furthermore, recent research highlights the critical role of controlling DES viscosity and pH to optimize mass transfer and preserve native-like protein conformations (Anvar et al., 2025; Moldes et al., 2024). Moreover, direct structural analyses are scarce in the context of DES-extracted hazelnut proteins, leaving questions about conformational integrity and preservation mechanisms unanswered. These knowledge gaps limit the development of scalable, green extraction technologies for high-value protein recovery from oilseed residues.

To address these gaps, the present study investigates the ultrasound-assisted extraction of hazelnut proteins using DES formulations and compares them to conventional alkaline extraction. The study comprehensively evaluates protein yield, purity, secondary structure, molecular weight distribution, and functional attributes (solubility, emulsifying activity, and foaming properties). By systematically comparing DES–US and alkaline methods, this work aims to define the extraction strategies for valorizing hazelnut meal in sustainable food systems, contributing to circular bioeconomy goals and reducing reliance on animal-derived proteins.

## 2 Materials and methods

### 2.1 Materials

Hazelnut meal, a by-product of cold-pressed hazelnut oil production, was kindly donated by Fiskobirlik (Giresun, Turkey). All chemicals used in the study were of analytical grade or higher and were purchased from Sigma-Aldrich (Steinheim, Germany). The protein content of the hazelnut meal and protein was determined according to standard AOAC methods (1990).

### 2.2 Synthesis of DESs

Deep eutectic solvents (DESs) were prepared by combining choline chloride (ChCl) with glycerol (polyol), sorbitol (sugar alcohol), or glucose (sugar). For each HBA–HBD pair, the molar ratio, ChCl:glycerol 1:2, ChCl:sorbitol 1:1, and ChCl:glucose 2:1, was selected from literature-reported stable eutectic compositions to ensure homogeneous, stable DES formation (Ozkan, 2024). As summarized in Table S1, mixtures were stirred at 80 °C for ∼2 h in sealed vessels until a clear, homogeneous liquid formed. DESs were then diluted with 40 % (*v*/v) distilled water to reduce viscosity and improve mass transfer while maintaining the eutectic hydrogen-bond network (Vilková et al., 2020). The prepared solutions were cooled to room temperature (∼22 °C) and characterized for pH (pH meter), viscosity (rotational rheometer), refractive index (refractometer), and density (pycnometer).

### 2.3 Protein extraction using deep eutectic and conventional alkaline solvents

The following methods were employed to extract protein from hazelnut meal:

i) *Protein extraction using DES:* For US-assisted extractions, hazelnut meal (∼30 g) was transferred into a beaker containing 600 mL of the corresponding DES formulation, maintaining a solid-to-solvent ratio of 1:20 (*w*/*v*). Extraction was performed using a probe-type ultrasonic processor (Sonics, 750 W, USA) equipped with a 13 mm tip, operating at 400 W, 30 % amplitude, in pulsed mode (5 s on / 5 s off) for 30 min. During extraction, the suspension temperature was controlled at 25 ± 5 °C with the aid of an ice bath. Following extraction, the mixtures were centrifuged at 13000 ×*g* for 20 min at 25 °C (Universal 320R, Hettich, Germany), and the supernatants were collected for protein quantification using the Bradford assay (Bradford, 1976). To induce protein precipitation, distilled water was added to the supernatant at volume ratios of 5:1 (*v*/v) relative to the DES volume. The mixtures were incubated overnight at 4 °C for cold antisolvent precipitation. Subsequently, the suspensions were centrifuged at 13000 ×*g* for 20 min at 4 °C to recover the protein precipitates. The pellets were washed three times with 40 mL of distilled water, followed by centrifugation at 13000 ×*g* and 4 °C for 10 min after each wash.
ii) *Protein extraction using the conventional alkaline method:* The alkaline extraction procedure was adapted from the method described by Karaca et al. (2011), with minor modifications. Ground hazelnut meal was suspended in distilled water at a solid-to-liquid ratio of 1:20 (*w*/*v*). The pH of the suspension was adjusted to 8.0, 9.0, or 10.0 using 5 M NaOH. The mixture was stirred at room temperature for 1 h to facilitate protein solubilization. Following extraction, the suspension was centrifuged at 13000 ×*g* for 20 min at 4 °C to separate the supernatant (Karabulut et al., 2024; Zhu et al., 2017). Protein precipitation was induced by adjusting the supernatant to pH 4.5 using 2 M HCl, followed by centrifugation at 13000 ×*g* for 20 min at 4 °C to recover the protein precipitates.

The final precipitates were freeze-dried and weighed to determine the extraction yield and efficiency. DES formulations included ChCl:glycerol (DES-GLY), ChCl:sorbitol (DES-SOR), and ChCl:glucose (DES-GLU). To evaluate the effect of US, parallel extractions were conducted under identical conditions without ultrasonic treatment; these control samples were designated as DES-GLY-C, DES-SOR-C, and DES-GLU-C, respectively. The samples extracted at different pH values (8.0, 9.0, and 10.0) were coded as ALK8, ALK9, and ALK10, respectively.

### 2.4 Determination of protein recovery and extraction efficiency

Protein yield and extraction efficiency were calculated at two distinct stages to comprehensively assess the extraction process. At the supernatant stage, following centrifugation of the extracts, the concentration of soluble protein was quantified using the Bradford assay according to Eqs. (1), (2). In the second stage, after protein precipitation and freeze-drying, the mass and purity of the dried protein isolates were used for calculations using the following Eqs. (3), (4):

Stage 1 — Soluble extract (before precipitation):

(1) $${\text{Yield}}_{\text{supernatant}}\;\left(\mathrm{mg}\;\text{protein}/g\;\text{meal}\right)=\frac{\mathrm{Cs}\times \mathrm{Vt}\;}{\mathrm{Wm}}$$

(2) $${\text{Extraction efficiency}}_{\text{supernatant}}\;\left(\%\right)=\frac{\mathrm{Cs}\times \mathrm{Vt}\;}{\mathrm{Po}}\times 100\;$$

Stage 2 — Powder/Isolate (after precipitation + drying):

(3) $${\text{Yield}}_{\text{powder}}\;\left(\mathrm{mg}\;\text{protein}/g\;\text{meal}\right)=\frac{\mathrm{Ap}\times \mathrm{Pp}\times 1000\;}{\mathrm{Wm}}\;$$

(4) $${\text{Extraction}}_{\text{powder}}\;\left(\%\right)=\frac{\mathrm{Ap}\times \mathrm{Pp}\times 1000\;}{\mathrm{Po}}\times 100\;$$

where Cs is the protein concentration in supernatant (mg/mL), Vt is the total extract volume (mL), Wm is the meal mass (g), Cm is the protein content of meal (mg protein/g meal); Po = Wm×Cm is the protein content in the meal (g), Pp is the protein purity of the powder as mass fraction, Apis the mass of dried isolate/powder (g).

### 2.5 **A**min**o acid profile**

Proteins were hydrolyzed in 6 M HCl at 110 °C for 24 h in sealed glass tubes. Prior to sealing, tubes were purged with nitrogen for ∼30 s to minimize oxidative degradation; hydrolysis was carried out in the presence of the kit antioxidant system supplied in Reagent-2 (Jasem Amino Acid Analysis Kit, Jasem, Germany). After cooling, hydrolysates were centrifuged (13,000 ×*g*, 5 min). An aliquot (100 μL) of the supernatant was diluted to 1 mL with water and serially diluted to yield an 800-fold dilution. For kit-based prep, 50 μL of the diluted hydrolysate was combined with 50 μL of a stable-isotope-labeled internal standard mix and 700 μL Reagent-1 (Jasem kit), vortexed (5 s), and transferred to vials for analysis.

Chromatography was performed on an Agilent 1260 Infinity HPLC with a Jasem amino acid analytical column (3.0 × 100 mm, particle size:3 μm, stationary phase:amine/weak−cation−exchange hybrid) maintained at 30 °*C. mobile* phase A was water with 0.1 % formic acid and 5 mM ammonium formate, and mobile phase B was acetonitrile with 0.1 % formic acid. A 7.5-min gradient was used at 0.7 mL min^−1^ (inj. Volume 3 μL): 0–0.5 min, 2 % B; 0.5–5.5 min, linear to 60 % B; 5.5–6.0 min, to 95 % B; 6.0–6.5 min, hold 95 % B; 6.5–7.5 min, re-equilibrate to 2 % B.

Detection used an Agilent 6460 triple quadrupole with ESI in positive mode (nitrogen, >99.999 % purity, as both nebulizer and drying gas; gas temp 150 °C; gas flow 10 L/min; nebulizer 40 psi; capillary +2000 V). Quantification employed multiple-reaction monitoring (MRM); transitions for each amino acid and the corresponding isotope-labeled internal standard, together with fragmentor voltages and collision energies, are listed in Table S2. Data were acquired with per-compound optimized dwell times (10–20 ms). Calibration used an external multi-analyte standard set normalized by the internal standards (isotopic-dilution). Seven-point curves [0.5–100 μM∗∗] were fitted by weighted linear regression (1/x), with acceptance criteria R^2^ ≥ 0.995 and back-calculated concentrations within ±15 % of nominal (±20 % at LLOQ). Quality controls at low/mid/high levels were included in each batch (Bilgin et al., 2019).

### 2.6 Sodium dodecyl sulfate polyacrylamide gel electrophoresis (SDS-PAGE)

The subunit composition and band distribution of the extracted proteins were analyzed using precast 4–20 % acrylamide gel cassettes (Genesuz, USA). Protein samples (3 mg/mL) were mixed with Sodium Dodecyl Sulfate **(**SDS) sample buffer and denatured at 95 °C for 5 min. Subsequently, 5 μL of each sample was loaded onto a Mini-PROTEAN electrophoresis system (Bio-Rad Laboratories, Hercules, CA, USA), along with a molecular weight marker (10–270 kDa). Electrophoresis was conducted at 180 V until the dye front reached the bottom of the gel. Protein bands were visualized by staining with 0.1 % Coomassie Brilliant Blue R-250 in 50 % methanol and 10 % acetic acid, followed by destaining overnight in 10 % acetic acid (He, 2011).

### 2.7 Fourier transform infrared spectroscopy (FTIR)

Structural changes in the extracted proteins, particularly in the Amide *I*, II, and III regions, were analyzed using Attenuated Total Reflectance-Fourier Transform Infrared (ATR-FTIR) spectroscopy (Thermo Electron Co., WI, USA). Prior to measurement, freeze-dried protein samples were finely ground and directly placed onto the ATR crystal to ensure good contact and minimize moisture interference. Spectra were recorded over the range of 400–4000 cm^−1^ at a resolution of 16 cm^−1^, with 120 scans averaged per sample. Background spectra were collected before each measurement and automatically subtracted.

### 2.8 Circular dichroism (CD) spectroscopy

CD spectroscopy was performed to evaluate secondary structure alterations in protein samples (0.25 mg/mL) at 25 °C, following the method described by Malomo et al. (2014). Prior to measurement, protein isolates were dissolved in phosphate buffer (10 mM, pH 7.4) and filtered through a 0.22 μm membrane to remove particulates. Spectra were recorded using a spectropolarimeter (Jasco J–715, MD, USA) with a 0.1 cm quartz cuvette over the wavelength range of 190–260 nm. Each spectrum was obtained by averaging three scans, with the baseline (buffer only) subtracted from the protein spectra. The resulting spectra were analyzed using the Beta Structure Selection (BeStSel) algorithm (https://bestsel.elte.hu/index.php) to estimate the distribution of secondary structural elements.

### 2.9 Intrinsic fluorescence emission

The intrinsic fluorescence emission of the protein samples was measured using a spectrofluorometer (Hitachi F7000, Tokyo, Japan). Protein suspensions (0.25 mg/mL) were prepared in 10 mM sodium phosphate buffer (pH 7.0) and analyzed at room temperature in a four-sided quartz cuvette. Fluorescence emission spectra were recorded with an excitation wavelength of 280 nm, and emission was scanned from 300 to 450 nm.

### 2.10 Scanning electron microscopy (SEM)

The morphological characteristics of the extracted protein isolates were examined using a scanning electron microscope (Jeol JSM 6060 LV, Japan). Prior to imaging, freeze-dried protein powders were gently ground to obtain fine particles and mounted onto aluminum stubs using double-sided carbon adhesive tape. The mounted samples were then sputter-coated with a thin layer of gold:palladium alloy (60:40) under vacuum to enhance surface conductivity and minimize charging effects during observation. SEM analyses were conducted in high-vacuum mode at an accelerating voltage of 15 kV, and images were captured at magnifications of 100× and 500 × .

### 2.11 Particle size and zeta potential

Particle size distribution and surface charge (zeta potential) of the protein samples were determined using a Mastersizer 2000 and Zetasizer Nano ZS (Malvern Instruments Ltd., UK). Prior to measurement, freeze-dried protein powders were re-dissolved in distilled water at a final protein concentration of 1.0 % (*w*/*v*) and the pH adjusted to 7.0 with 0.1 M NaOH or HCl. The suspensions were agitated for 5 min before analysis. Measurements were conducted at room temperature (∼22 °C) using refractive index values of 1.45 for the protein, 0.10 for absorption, and 1.33 for the dispersant (water).

### 2.12 Analysis of techno-functional and nutritional properties

#### 2.12.1 Solubility

Protein solubility was determined using the modified Bradford assay (Bradford, 1976). Briefly, protein samples (5 mg/mL in distilled water) were centrifuged at 13,000 ×*g* for 25 min at 25 °C, and the resulting supernatants were collected. An aliquot of 150 μL from each supernatant was mixed with 3 mL of Bradford reagent, and the mixtures were incubated for 10 min at room temperature. Absorbance was measured at 595 nm using a UV–Vis spectrophotometer (Shimadzu UV-1240, Japan). Distilled water was used as the blank. A standard calibration curve was constructed using bovine serum albumin (BSA) (y = 0.0016× + 0.0214, *R*^2^ 0.9952) (Eq. 5).

(5) $$\text{Protein solubility}\;\left(\%\right)=\frac{\text{Soluble protein content}}{\text{Total protein content}\;}\times 100$$

#### 2.12.2 Emulsifying properties

Emulsifying properties were assessed according to the method of Pearce and Kinsella (1978). Oil-in-water (o/w) emulsions were prepared by adding 1 mL of sunflower oil to 3 mL of protein solution, resulting in a final oil concentration of 0.25 % (*w*/w). The mixtures were homogenized using an Ultra-Turrax homogenizer (IKA, T18, Königswinter, Germany) at 10,000 rpm for 2 min. The absorbance of the freshly prepared emulsions was measured at 500 nm immediately (A₀) and after 10 min (A₁₀) using a UV–Vis spectrophotometer. The emulsion activity index (EAI) and emulsion stability index (ESI) were calculated using Eqs. (6), (7), respectively.

(6) $$\mathrm{EAI}\;\left(m^{2}/g\right)=\frac{\left(2\times 2,303\times A₀x\;\mathrm{SF}\right)\;}{\left(C\times \theta \;\times \Phi \times 10000\right)}$$

(7) $$\mathrm{ESI}\;\left(\min.\right)=\frac{\left(A₀\times \mathrm{\Delta t}\right)\;}{\left(A₀-A₁₀\right)}$$

where SF is the dilution factor, C is the weight of protein per unit volume (g/mL), θ is the optical path width (0.01 m), Φ is the oil volume fraction.

#### 2.12.3 **Foa**min**g properties**

The foaming properties of the protein isolates were evaluated according to the method of Karabulut and Feng (2024), with minor modifications. A 10 mL aliquot of protein suspension (5 mg/mL) was subjected to vigorous mixing at room temperature using a high-speed homogenizer (IKA, T18, Königswinter, Germany) operating at 10,000 rpm for 2 min. The total volume of the foam was recorded immediately after mixing and subsequently after standing for 15, 30, 60, 90, and 120 min at room temperature. Foaming capacity (FC) and foam stability (FS) were calculated using the corresponding Eqs. (8), (9):

(8) $$\mathrm{FC}\;\left(\%\right)=\left(\frac{V_{0}-V}{V}\right)\times 100$$

(9) $$\mathrm{FS}\;\left(\%\right)=\left(\frac{V_{t}-V}{V_{0}-V}\right)\times 100$$

where V is the initial volume before whipping (mL), $V_{0}$ is the volume of the suspension after homogenization (mL), $V_{t}$ is the total volume immediately after homogenization t time (mL).

### 2.13 In-vitro digestibility

The in vitro digestion model developed by the INFOGEST network (Minekus et al., 2014) was applied to simulate sequential gastric and intestinal phases. Briefly, 5 mL of the beverage sample was mixed with 4 mL of simulated salivary fluid, 25 μL of 0.3 M CaCl₂, and 975 μL of distilled water, then preincubated at 37 °C and 100 rpm for 2 min. For the gastric phase, 7.5 mL of simulated gastric fluid, 1.6 mL of pepsin (25,000 U/mL), and 5 μL of CaCl₂ were added; the pH was adjusted to 3 with 1 M HCl, and the volume was brought to 20 mL with water before incubation at 37 °C for 2 h. Subsequently, 5 mL aliquots were collected, and intestinal digestion was initiated by adding 8.25 mL of simulated intestinal fluid, 3.75 mL of pancreatin (800 U/mL), 1.875 mL of bile (160 mM), and 30 μL of CaCl₂. The pH was adjusted to 7 with 1 M NaOH, and the volume was completed to 30 mL before incubation at 37 °C for another 2 h. A blank digestion without the sample was included to correct for background interferences. Digested samples were cooled in an ice bath, centrifuged at 13000 ×*g* for 30 min at 4 °C, and the supernatants (bioaccessible fractions) were analyzed immediately.

### 2.14 Statistical analysis

All analyses were performed in triplicate, and the results are presented as mean ± standard deviation. Statistical evaluations were conducted using the SPSS 20.0 software package (SPSS Inc., Chicago, USA). One-way analysis of variance (ANOVA) was applied to determine significant differences among sample groups, followed by Duncan's multiple range test for post-hoc comparisons at a 95 % confidence level (*p* < 0.05).

## 3 Results and discussion

### 3.1 Protein extraction performance and influence of DES physicochemical properties

**Fig. 1:**
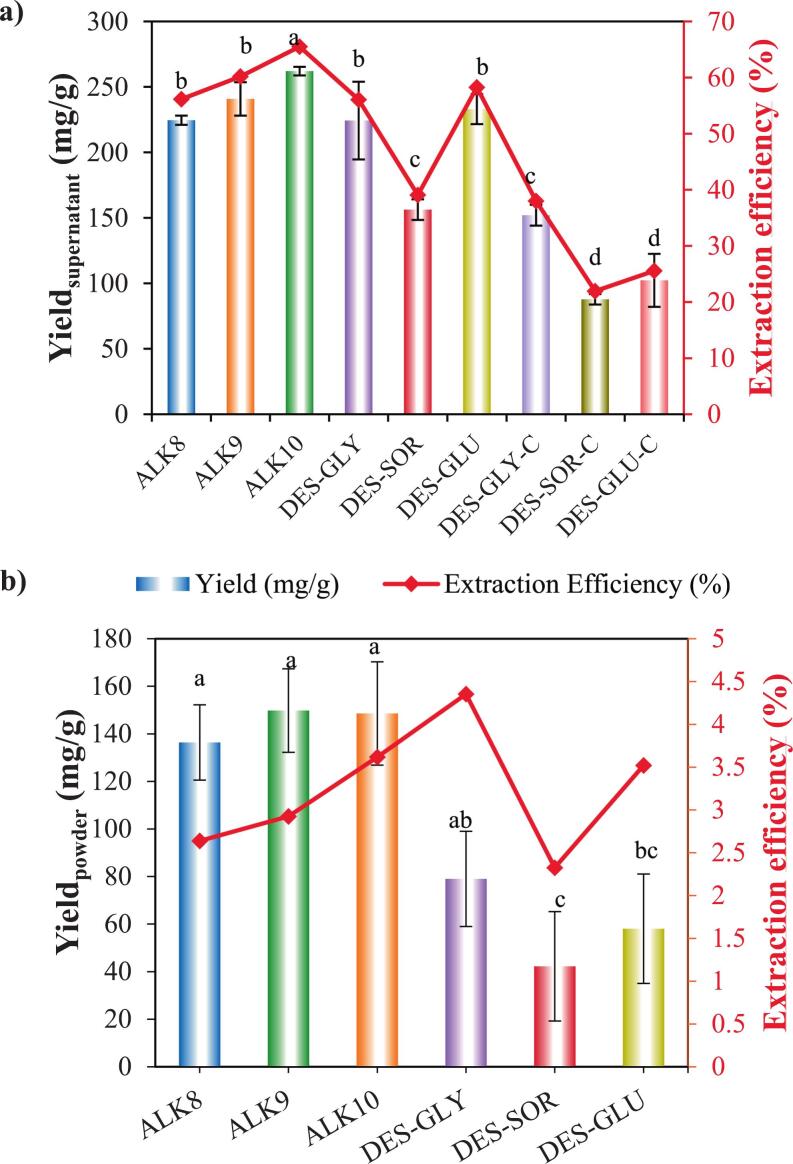
Yield (mg/g) and extraction efficiency (%) of alkaline and DES-extracted hazelnut proteins a) in supernatant and b) powder form.

Protein extraction yield and efficiency were systematically assessed at two key stages: (i) the soluble supernatant before freeze-drying (Fig. 1a), reflecting solubilization performance, and (ii) the recovered dried isolates (Fig. 1b), representing practical recoverability. ALK10 treatment delivered the highest yield (∼260 mg/g) and efficiency (∼63 %), significantly outperforming all other conditions (*p* < 0.05). This superior extraction is attributed to elevated pH promoting deprotonation of acidic side chains, increasing net negative charge and electrostatic repulsion that disrupts protein–cell wall and matrix interactions, thereby enhancing solubilization (Karimi et al., 2024; Malomo et al., 2014). ALK9 and ALK8 treatments, with slightly lower pH, still maintained efficiencies above 55 %, confirming the pH-dependent modulation of protein solubility via ionic interactions and matrix swelling. Such pH effects are consistent with reports for canola and rapeseed meals, where higher pH improves solubilization but risks structural denaturation (Đermanović et al., 2025; Karimi et al., 2024).

Among DES-based systems, US-assisted extractions with DES-GLY and DES-GLU achieved moderate yields (∼200–220 mg/g) and efficiencies of 50–55 %. These results highlight the capacity of DES to disrupt non-covalent interactions (hydrogen bonding, hydrophobic associations) in the cell wall matrix, while cavitation-driven microjets and shear forces of US enhance mass transfer and overcome the intrinsic viscosity limitations of DES media (Bayram et al., 2024; Chemat et al., 2016; Lores et al., 2017). However, DES-SOR formed a stable eutectic system, its intermediate viscosity (97.8 mPa·s) and strong hydrogen-bonding capacity likely limited protein diffusion and hindered efficient precipitation. In addition, residual sorbitol molecules may have interacted with protein side chains, leading to partial shielding of functional groups and increased aggregation during the recovery stage. These factors collectively contributed to the lower yield observed for DES–SOR compared to DES–GLY and DES–GLU.

Physicochemical properties of the DES systems (Table S1) critically influenced outcomes. DES-GLY, with the lowest viscosity (56.3 mPa·s) and moderate polarity, promoted better solute–solvent interactions and molecular mobility, enabling more efficient extraction. DES-SOR displayed intermediate behavior (97.8 mPa·s), reinforcing the direct relationship between solvent rheology and extraction efficiency. By contrast, the viscosity of DES–GLU (181.4 mPa·s) was nearly twofold higher than DES–GLY and DES–SOR, which imposed stronger mass transfer limitations. This hindered solvent penetration and protein diffusion during the precipitation step, thereby explaining the markedly lower recovery observed for DES–GLU (Anvar et al., 2025; Chakravorty & Das, 2025). Such viscosity effects are well documented; for instance, ChCl–oxalic acid systems, despite high acidity, showed limited mass transfer due to viscosity in sesame meal extractions (Cao et al., 2023).

Post-freeze-drying analysis (Fig. 1b) showed yield declines across all treatments, attributed to moisture removal, aggregation, and conformational destabilization during freeze–thaw. Alkaline treatments retained the highest dried yields (130–150 mg/g), indicating efficient protein precipitation and minimal structural change. In contrast, DES-based systems exhibited more pronounced yield losses: DES-GLY retained ∼80 mg/g (∼3.5 % efficiency), while DES-SOR and DES-GLU fell below 60 mg/g and 3 %, likely due to strong DES–protein interactions and high viscosity hindering precipitation and promoting aggregation (Ruesgas-Ramón et al., 2017; Zhu et al., 2017). Similar challenges have been observed in rice dreg and cassava leaf extractions (Patra & Venugopal, 2025; Shahid et al., 2024). Although DES–US extractions exhibited promising solubilization performance, the actual recovery rates after lyophilization were relatively low. This limitation can be attributed to the strong hydrogen-bonding interactions between DES components and protein side chains, as well as the higher viscosity of the extraction media, both of which hinder efficient precipitation and favor aggregation during recovery. To overcome these challenges, optimization of downstream steps is crucial. For instance, optimized antisolvent precipitation conditions (e.g., solvent-to-water ratio, temperature, and incubation time) may improve protein release and minimize aggregation. Alternatively, membrane-based separation techniques such as ultrafiltration or dialysis could be employed to facilitate solvent removal and protein concentration while preserving structural integrity. Incorporating such strategies may significantly enhance the practical recovery of proteins from DES–US systems and improve their applicability in large-scale processes.

### 3.2 Amino acid profile of hazelnut proteins

Analysis of amino acid composition (Table 1) highlights clear differences in extraction performance driven by solvent environment and physical assistance. DES–US extractions consistently yielded higher total essential amino acid (EAA) contents than alkaline treatments. DES-SOR achieved the highest EAA level (22.66 mg/g), followed by DES-GLY (21.54 mg/g) and DES-GLU (21.06 mg/g). In contrast, alkaline treatments produced significantly lower EAA totals, with ALK9 at 19.24 mg/g, ALK8 at 18.37 mg/g, and ALK10 at only 15.94 mg/g. This advantage of DES–US systems in preserving EAA content aligns with findings for canola, rapeseed, and sesame meals, where milder DES conditions limit harsh side-chain modifications (Cao et al., 2023; Đermanović et al., 2025; Karimi et al., 2024).

**Table 1:** Amino acid composition of hazelnut protein extracted using classic alkaline and DES-based methods.

**Aminoacids (mg/g)**						
**Essential amino acids**	**ALK8**	**ALK9**	**ALK10**	**DES-GLY**	**DES-SOR**	**DES-GLU**
Valine	2.49 ± 0.02^d^	2.59 ± 0.02^c^	2.11 ± 0.03^e^	3.23 ± 0.06^a^	3.19 ± 0.02^a^	3.05 ± 0.02^b^
Leucine	4.30 ± 0.03^c^	4.32 ± 0.03^c^	3.76 ± 0.05^d^	4.99 ± 0.04^b^	5.25 ± 0.09^a^	5.30 ± 0.01^a^
Isoleucine	1.42 ± 0.05^d^	1.46 ± 0.05^d^	1.28 ± 0.00^e^	1.91 ± 0.00^a^	1.73 ± 0.01^c^	1.81 ± 0.01^b^
Threonine	2.38 ± 0.07^c^	2.48 ± 0.06^c^	1.94 ± 0.05^d^	2.72 ± 0.02^b^	2.98 ± 0.15^a^	2.51 ± 0.01^c^
Methionine	0.50 ± 0.01^c^	0.63 ± 0.01^a^	0.44 ± 0.01^e^	0.49 ± 0.01^c^	0.57 ± 0.00^b^	0.47 ± 0.01^d^
Phenylalanine	3.65 ± 0.07^d^	3.76 ± 0.07^d^	3.28 ± 0.06^e^	4.36 ± 0.00^b^	4.63 ± 0.04^a^	4.08 ± 0.03^c^
Lysine	1.80 ± 0.00^c^	2.04 ± 0.03^b^	1.47 ± 0.01^d^	1.80 ± 0.02^c^	2.15 ± 0.03^a^	1.87 ± 0.06^c^
Histidine	1.83 ± 0.04^d^	1.96 ± 0.04^c^	1.65 ± 0.02^e^	2.04 ± 0.01^b^	2.16 ± 0.02^a^	1.97 ± 0.01^c^
**Total**	18.37 ± 0.02^e^	19.24 ± 0.03^d^	15.94 ± 0.13^f^	21.54 ± 0.08^b^	22.66 ± 0.06^a^	21.06 ± 0.01^c^
**Non-essential amino acids**						
Alanine	3.86 ± 0.03^d^	3.90 ± 0.01^d^	3.32 ± 0.01^e^	4.28 ± 0.04^c^	4.74 ± 0.03^a^	4.58 ± 0.00^b^
Glycine	2.05 ± 0.00^d^	2.79 ± 0.01^c^	2.55 ± 0.10^c^	3.77 ± 0.08^b^	4.02 ± 0.10^b^	5.12 ± 0.29^a^
Aspartic acid	9.71 ± 0.01^d^	8.30 ± 0.01^e^	7.03 ± 0.01^f^	11.83 ± 0.04^a^	11.16 ± 0.04^c^	11.23 ± 0.00^b^
Glutamic acid	15.85 ± 0.11^e^	17.55 ± 0.37^d^	14.91 ± 0.10^e^	22.53 ± 0.03^a^	21.42 ± 0.02^b^	20.15 ± 0.21^c^
Ornithine	0.06 ± 0.00^c^	0.06 ± 0.00^c^	0.06 ± 0.00^d^	0.08 ± 0.00^a^	0.08 ± 0.00^b^	0.08 ± 0.00^b^
Proline	3.07 ± 0.01^d^	3.17 ± 0.01^c^	2.60 ± 0.01^e^	3.48 ± 0.06^b^	3.87 ± 0.03^a^	3.53 ± 0.02^b^
Serine	4.26 ± 0.07^c^	3.84 ± 0.03^d^	3.25 ± 0.00^e^	4.63 ± 0.00^b^	5.11 ± 0.02^a^	4.56 ± 0.01^b^
Tyrosine	1.66 ± 0.02^b^	1.76 ± 0.00^b^	1.65 ± 0.02^b^	2.08 ± 0.11^a^	2.12 ± 0.04^a^	2.18 ± 0.03^a^
Cystine	0.81 ± 0.02^c^	0.68 ± 0.01^d^	0.61 ± 0.03^e^	1.11 ± 0.01^a^	1.14 ± 0.01^a^	1.05 ± 0.02^b^
Arginine	10.36 ± 0.03^d^	9.62 ± 0.01^e^	8.56 ± 0.02^f^	13.09 ± 0.02^b^	13.72 ± 0.04^a^	12.88 ± 0.00^c^
**Total**	51.70 ± 0.01^c^	51.68 ± 0.41^c^	44.55 ± 1.00^d^	66.89 ± 0.29^a^	67.38 ± 0.05^a^	65.35 ± 0.08^b^

Data are expressed as a mean ± standard deviation of three replicate (*n* = 3). Different characters in the same row were notably distinct between samples (*p* < 0.05).

DES systems leverage extensive hydrogen-bond donor/acceptor networks that stabilize protein structures during solubilization, mitigating harsh denaturation and side-chain modification typical of strong alkali conditions (Anvar et al., 2025; Moldes et al., 2024). US further enhances extraction via cavitation-driven microjets that disrupt cell walls and improve mass transfer, while avoiding the uncontrolled unfolding that risks amino acid loss (Chemat and Khan, 2011; Bayram et al., 2024).

This preservation is also evident in non-essential amino acid (NEAA) profiles. DES-extracted samples displayed higher NEAA totals (∼65–67 mg/g), with DES-SOR (67.38 mg/g) and DES-GLY (66.89 mg/g) outperforming alkaline treatments (44.55–51.70 mg/g). Similar enhancements were reported by Anvar et al. (2025) and Moldes et al. (2024). In rice bran and cassava leaf proteins, NADES treatments likewise preserved higher NEAA levels while improving solubility and functional properties (Patra & Venugopal, 2025; Shahid et al., 2024).

Key amino acids such as glutamic acid and arginine were significantly enriched in DES-treated isolates. For instance, DES-GLY contained 22.53 mg/g glutamic acid, versus 14.91 mg/g in ALK10. Arginine levels similarly favored DES-SOR (13.72 mg/g) over ALK10 (8.56 mg/g). These amino acids are critical for solubility, buffering capacity, and interfacial activity, and have health-promoting roles such as umami taste enhancement and cardiovascular benefits (Cao et al., 2023; Chen et al., 2025). By contrast, alkaline extraction relies on strong pH-induced denaturation. Elevated pH leads to deprotonation of acidic side chains, increasing net negative charge and disrupting ionic and hydrogen-bonded interactions, which enhances matrix dissolution but can also result in side-chain modifications, cleavage, or loss of labile residues (Hanafi et al., 2024). Studies on rapeseed and canola highlight that while alkaline treatments maximize yield, they risk compromising amino acid composition through deamidation and β-elimination reactions (Đermanović et al., 2025; Karimi et al., 2024).

### 3.3 Molecular bands of hazelnut proteins

**Fig. 2:**
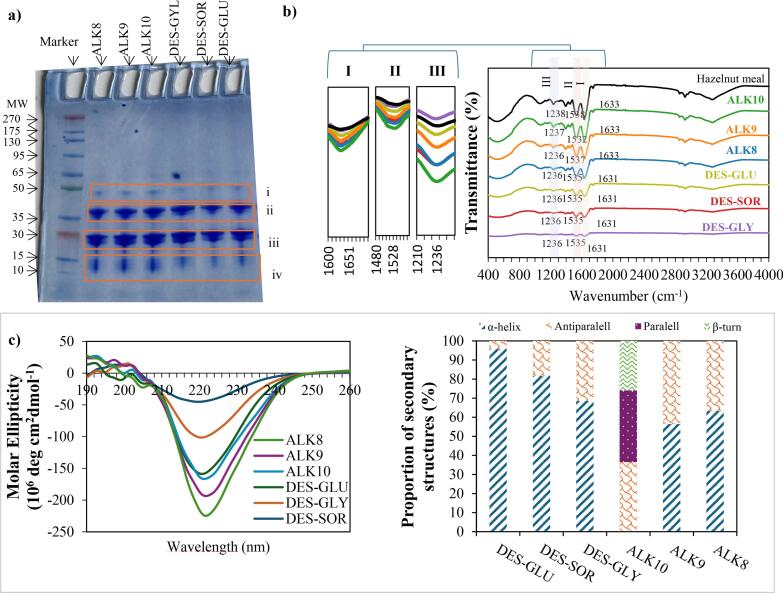
a) SDS-PAGE protein bands under reducing conditions, b) FTIR spectra, and c) CD spectroscopy and the proportion of secondary structure of alkaline and DES-extracted hazelnut proteins.

SDS-PAGE analysis under reducing conditions was used to evaluate the molecular integrity and subunit composition of hazelnut proteins (Fig. 2a). All samples exhibited characteristic bands for nut storage proteins: vicilin-type 7S globulins (∼35–50 kDa), legumin-type 11S globulins (acidic ∼35–40 kDa; basic ∼20–30 kDa), and smaller 2S albumins (∼10–15 kDa) (Barre et al., 2008; Ceylan et al., 2023).

Profiles of alkaline- and DES–US-extracted proteins were notably similar, with no major shifts in band positions or subunit loss, demonstrating effective recovery of dominant storage proteins in both approaches (Anvar et al., 2025; Bayram et al., 2024). This aligns with previous work showing that DES extraction maintained band intensities and patterns comparable to alkali controls, indicating minimal degradation or subunit loss (Wang et al., 2025). Notably, these results highlight the ability of DES to stabilize protein structures even under mechanical disruption, supporting high-quality protein recovery.

Additionally, the consistent intensity of 2S albumin and globulin bands in all treatments reflects the inherent stability of these disulfide-rich proteins, which resist denaturation and remain soluble under various processing conditions (Arulrajah et al., 2025; Cao et al., 2023). DES systems may further aid this stability by forming strong hydrogen-bonding networks that help maintain disulfide linkages and reduce non-specific aggregation (Karabulut et al., 2024; Patra & Venugopal, 2025). The protective effect of DESs on disulfide bonds can be explained by their extensive hydrogen-bonding networks. The hydrogen bond donors and acceptors within DESs interact with polar amino acid side chains in the vicinity of cysteine residues, thereby creating a stabilizing microenvironment around disulfide linkages. This network reduces the accessibility of thiol groups to reductive or oxidative agents and minimizes conformational unfolding that could otherwise expose disulfide bonds to cleavage. Moreover, the high polarity of DES systems enhances the formation of hydrogen-bond-mediated hydration shells, which further restricts bond disruption. Similar stabilization mechanisms have been reported for sesame and zein proteins, where DESs maintained disulfide-rich structures by preventing aggregation and irreversible unfolding (Anvar et al., 2025; Cao et al., 2023). Briefly, these interactions explain how DESs contribute to the preservation of native disulfide bridges during extraction. These findings suggest that DES extraction preserves the dominant subunit composition of hazelnut proteins while minimizing chemical modifications like deamidation or oxidative cross-linking often seen with harsh alkaline conditions (Hanafi et al., 2024). Maintaining such molecular integrity is crucial for functional properties like solubility, emulsification, and foaming, which depend on intact, native-like subunit structures.

### 3.4 FTIR analysis of structural changes in hazelnut proteins

FTIR spectroscopy (Fig. 2b) was employed to analyze secondary structural features of hazelnut proteins, focusing on the Amide I (1600–1700 cm^−1^), Amide II (1480–1570 cm^−1^), and Amide III (1200–1300 cm^−1^) regions, which reveal backbone conformation and hydrogen-bonding patterns (Mundada et al., 2024).

In the Amide I region (∼1631–1633 cm^−1^), alkaline-extracted proteins, especially ALK10, showed sharper, more intense peaks, indicative of well-defined β-sheet structures and strong intramolecular hydrogen bonding. Conversely, DES–US-extracted samples exhibited broader, less shifted Amide I and II bands, indicating more preserved, native-like secondary structures. This stabilization is attributed to the strong hydrogen-bond donor–acceptor networks of DES solvents that help maintain protein folding even under ultrasonic treatment (Chen et al., 2025; Moldes et al., 2024). Such mild preservation effects have been consistently documented in sesame, peanut meal, and mushroom stem extractions using DES, where denaturation and extensive unfolding were limited (Cao et al., 2023; Jiang et al., 2024; Karabulut et al., 2024). US combination enhances mechanical disruption and extraction efficiency without significantly increasing the denaturing potential of DES. Instead, US–DES processes achieve partial, controlled unfolding that exposes functional groups while maintaining overall secondary structure integrity, as a key factor for preserving solubility and emulsification properties (Anvar et al., 2025; Bayram et al., 2024; Hanafi et al., 2024). Additionally, shifts and broadening in the O—H stretching region (3200–3400 cm^−1^) in DES-treated samples suggest strong solvent–protein hydrogen bonding and residual DES interactions. Such spectral features are consistent with other choline chloride-based DES extractions of plant proteins, where extensive hydrogen-bonding networks between solvent components and protein functional groups stabilize conformation and limit aggregation (Tjalsma et al., 2025).

### 3.5 Secondary structure profile by CD spectra

CD spectroscopy (Fig. 2c) offered detailed insights into the secondary structure of hazelnut proteins, with BeStSel deconvolution revealing marked differences in α-helix and β-sheet content across treatments, highlighting the influence of pH and solvent environment on protein conformation.

ALK10 extracts exhibited the lowest α-helix content and high antiparallel β-sheet contribution (36.5 %), indicating strong denaturation and refolding into β-rich structures. ALK9 and ALK8 similarly showed reduced α-helix levels (56.4 % and 63.3 %) with elevated β-sheet content (43.6 % and 36.7 %). These findings are consistent with known effects of high pH, where extensive unfolding disrupts intramolecular hydrogen bonds and promotes β-sheet-rich rearrangements, an established phenomenon in alkaline-extracted legume and oilseed proteins (Arulrajah et al., 2025; Cao et al., 2023; Đermanović et al., 2025; Karimi et al., 2024; Wang et al., 2025).

By contrast, DES-extracted proteins showed clear differences depending on DES composition. DES-GLU and DES-SOR maintained exceptionally high α-helix content (95.7 % and 81.6 %), suggesting strong preservation of native-like helical domains despite the mechanical effects of US. This preservation is attributed to the hydrogen-bond donor capabilities of glucose and sorbitol, which promote stabilizing interactions with the polypeptide backbone, limiting irreversible unfolding (Anvar et al., 2025; Patra & Venugopal, 2025). Similar stabilization has been observed in mushroom stem and rice bran extractions, where DES treatments prevented the β-sheet enrichment typically induced by strong alkaline conditions (Karabulut et al., 2024; Shahid et al., 2024). In contrast, DES-GLY showed a distinct profile with reduced α-helix content (68.5 %) and higher antiparallel β-sheet contribution (31.5 %). Such differences likely arise from variations in viscosity, hydrogen-bond strength, and solvent–protein interactions (Hewage et al., 2024).

### 3.6 Colloidal properties and particle size distribution of hazelnut proteins

**Fig. 3:**
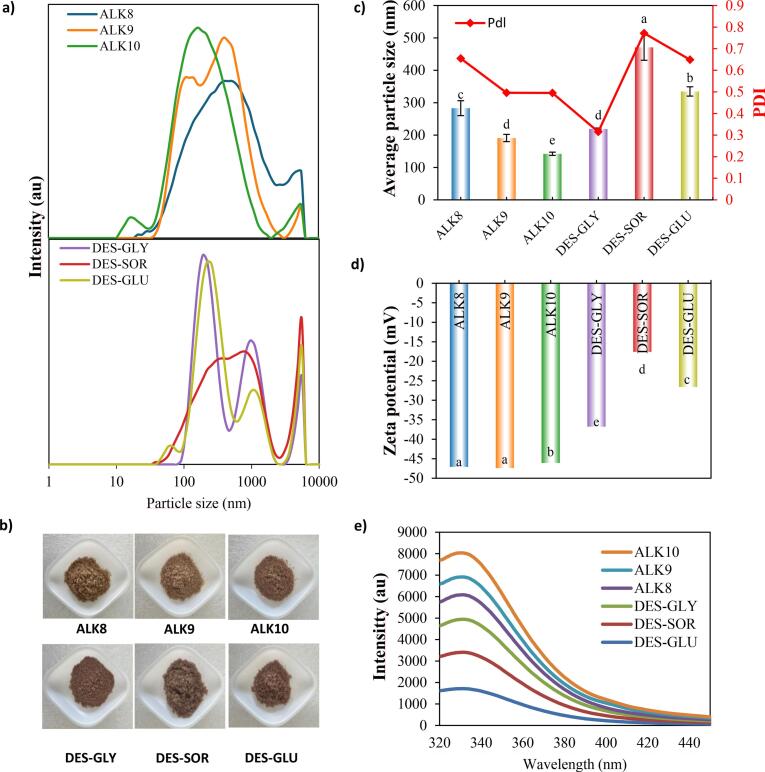
a) Particle distribution, b) Average particle size/particle distribution index (PDI), c) Powder forms, d) Zeta potential, and e) Intrinsic fluorescence spectra of alkaline and DES-extracted hazelnut proteins.

The colloidal properties of hazelnut protein isolates were evaluated via particle size distribution and polydispersity index (PDI) measurements (Fig. 3a–3c), providing insights into aggregation, dispersion uniformity, and functional behavior in solution. Fig. 3b illustrates the powder forms of hazelnut proteins extracted using alkaline and DES systems.

For alkaline-extracted samples, a clear pH-dependent reduction in particle size was observed: ALK10 showed the smallest mean size (∼140 nm), followed by ALK9 (∼190 nm) and ALK8 (∼280 nm) (Fig. 3c). This trend reflects high pH promoting protein unfolding and disaggregation by increasing net negative charge, enhancing electrostatic repulsion, and breaking non-covalent interactions within aggregates (Arulrajah et al., 2025). The unimodal, narrow distribution of ALK10 suggests a homogeneous, monodisperse population with efficient solvation and reduced protein–protein interactions (Fig. 3a). Such behavior mirrors findings in rapeseed and canola protein extractions, where high-pH treatments yielded well-dispersed, soluble fractions (Cao et al., 2023; Đermanović et al., 2025). In contrast, DES-extracted proteins exhibited larger, more heterogeneous particle size profiles. DES-SOR had the highest average size (∼500 nm), indicating limited solubility and strong hydrogen-bond networks hindering complete dissociation of high-molecular-weight fractions. DES-GLU and DES-GLY displayed intermediate sizes (∼360 nm and ∼ 220 nm, respectively) with broad, often bimodal distributions, signaling the coexistence of well-dispersed small particles and larger aggregates due to incomplete matrix disruption and less efficient solubilization compared to alkaline extraction (Anvar et al., 2025). Similar patterns have been reported for sesame and peanut meal DES extracts, where high viscosity and strong solvent–protein interactions limited full disaggregation (Cao et al., 2023).

PDI measurements reinforced these observations (Fig. 3c). ALK10 had the lowest PDI (∼0.49), reflecting narrow, uniform size distributions and stable suspensions. ALK9 and ALK8 also maintained low PDI values, supporting their well-dispersed character. By contrast, DES-extracted samples generally exhibited higher PDI values, with DES-SOR reaching ∼0.78, indicative of broader, polydisperse populations prone to aggregation. Notably, DES-GLY achieved the lowest PDI among DES systems (∼0.40), suggesting improved dispersion stability. Such improvements with ChCl:Gly systems have also been observed in mushroom stem and cassava leaf extractions, where glycerol-based DES promoted better particle break-up and reduced polydispersity (Karabulut et al., 2024; Patra & Venugopal, 2025). These results align with SEM, FTIR, and fluorescence analyses, highlighting how the extraction environment governs both molecular structure and colloidal behavior.

### 3.7 Zeta potential and surface charge properties of hazelnut proteins

Zeta potential measurements characterized the surface charge and electrostatic stability of hazelnut protein isolates from alkaline and DES extraction systems. This parameter is key to understanding colloidal behavior: higher absolute zeta potential values (more negative or positive) indicate stronger electrostatic repulsion between particles, reducing aggregation and enhancing dispersion stability (Salopek et al., 1992).

As shown in Fig. 3d, all samples exhibited negative zeta potentials at near-neutral pH, reflecting deprotonated acidic residues such as glutamic and aspartic acid on protein surfaces. Among alkaline extracts, ALK8 and ALK9 had the most negative zeta potentials (−45 mV), with ALK10 slightly less negative (−43 mV). These high negative values suggest robust electrostatic repulsion and stable dispersion, attributed to pH-induced deprotonation of acidic side chains during extraction. Alkaline conditions promote matrix disruption and protein unfolding, exposing and ionizing carboxyl groups to increase net negative charge density, a trend consistent with previous studies (Hanafi et al., 2024).

In contrast, DES-extracted proteins exhibited significantly less negative zeta potentials. DES-GLU and DES-GLY samples measured around −30 mV, while DES-SOR had the least negative value (−25 mV). This reduction can be explained by extensive hydrogen bonding from DES components (sugars and polyols) with protein side chains, which can partially shield or neutralize exposed charges (Anvar et al., 2025; Moldes et al., 2024). Additionally, the inherent viscosity of DES systems may reduce ionic mobility and create local ionic shielding effects that attenuate measurable zeta potential.

### 3.8 Intrinsic fluorescence spectra of hazelnut proteins

Intrinsic fluorescence spectroscopy was used to probe tertiary structural changes in hazelnut proteins by monitoring the environment-sensitive emission of aromatic residues, primarily tryptophan (Trp), as indicators of folding, core packing, and solvent exposure (Condict & Kasapis, 2022; Sengupta, 2021).

As shown in Fig. 3e, alkaline-extracted proteins, particularly ALK10, exhibited the highest fluorescence intensities (FI), indicating greater Trp exposure to the polar aqueous environment. This suggests partial unfolding and loosening of tertiary structure under high pH, which promotes deprotonation of acidic side chains, disrupts stabilizing intramolecular bonds, and increases electrostatic repulsion, yielding more flexible, solvent-accessible conformations (Arulrajah et al., 2025; Cao et al., 2023). ALK9 and ALK8 showed similarly elevated FI values, reinforcing this trend. Comparable pH-induced unfolding and Trp exposure have been reported in rapeseed, canola, and cottonseed protein extractions, where alkaline conditions led to partial denaturation and enhanced solvent accessibility (Đermanović et al., 2025; Karimi et al., 2024; Wang et al., 2025).

In contrast, DES-extracted proteins (DES-GLY, DES-SOR, DES-GLU) exhibited significantly lower fluorescence intensities, suggesting more compact tertiary structures with buried Trp residues. The strong hydrogen-bond donor–acceptor networks of DES systems help stabilize protein folding by maintaining tighter packing and limiting hydrophobic core exposure (Chen et al., 2025). Similar intrinsic fluorescence analyses have shown that DES-extracted proteins retain native-like tertiary packing and reduced aromatic residue exposure (Cao et al., 2023).

These findings are consistent with the FTIR and CD results, confirming that DES systems preserve both secondary and tertiary structures, limiting extensive unfolding, whereas alkaline extraction promotes solubilization at the cost of partial denaturation. Such coordinated preservation of structure in DES-derived proteins is known to improve functional properties like solubility, emulsification, and foam stability across oilseed and by-product matrices (Karabulut et al., 2024). Balancing solubilization and structural preservation is thus critical for designing sustainable extraction processes that yield high-quality, functional protein ingredients for food applications.

### 3.9 Morphological properties

**Fig. 4:**
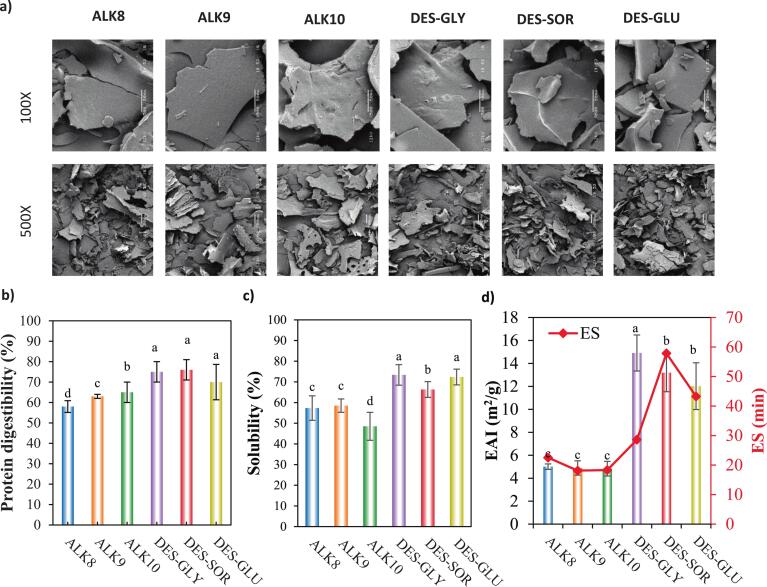
a) Morphological images using SEM at 100× and 500×, b) In vitro protein digestibility (%), c) Solubility (%), and d) Emulsion activity index (EAI)/Emulsion stability (ES) of alkaline and DES-extracted hazelnut proteins.

SEM imaging (Fig. 4a) was used to visualize the microstructural organization of hazelnut proteins, providing direct evidence of surface topology, aggregation behavior, and structural compactness under different extraction conditions.

Alkaline-extracted proteins (ALK8, ALK9, ALK10) exhibited dense, continuous, and compact morphologies at higher magnifications (500 × −1000×), reflecting strong protein–protein interactions and reaggregation during precipitation and drying. High pH promotes unfolding by deprotonating acidic side chains and exposing hydrophobic domains, which upon neutralization and drying reassociate via hydrophobic interactions and hydrogen bonds into cohesive, low-porosity matrices (Karabulut et al., 2024; Malomo et al., 2014). Such dense structures have also been reported for rapeseed, canola, and cottonseed proteins recovered at high pH, where extensive reaggregation leads to compact, less porous morphologies (Đermanović et al., 2025; Karimi et al., 2024; Wang et al., 2025). Interestingly, ALK10 showed slightly more porous and discontinuous domains than ALK8 and ALK9, suggesting that at pH 10 there is a subtle shift in the balance between solubilization and reaggregation, consistent with its improved solubility and dispersibility (Cao et al., 2023).

In contrast, DES-extracted proteins exhibited fragmented, flaky, and highly porous microstructures, particularly at lower magnifications (100 × −250×). DES-GLY, DES-SOR, and DES-GLU all displayed less cohesive morphologies with loosely associated particles and irregular edges. Similar porous, fragmented morphologies have been reported for DES systems, where hydrogen-bonding interactions prevent tight packing (Cao et al., 2023; Jiang et al., 2024; Karabulut et al., 2024).

DES formulations form extensive hydrogen bonds with protein functional groups, stabilizing partially unfolded states while preventing collapse into dense aggregates (Anvar et al., 2025). Such disruption of conventional aggregation leads to highly porous structures that can improve functional properties like solubility and emulsification (Patra & Venugopal, 2025).

### 3.10 Analysis of in-vitro digestibility and techno-functional properties

#### 3.10.1 In vitro digestibility

The in vitro digestibility of hazelnut protein isolates was significantly influenced by the extraction method (Fig. 4b, *p* < 0.05), underscoring the role of process-induced structural modifications in enzyme accessibility. DES-extracted proteins consistently demonstrated higher digestibility than alkaline-extracted samples. DES-SOR and DES-GLY achieved the highest values (∼76.0 % and ∼ 75.1 %), with DES-GLU also elevated (∼70.2 %). In contrast, alkaline-extracted proteins showed lower digestibility: ALK10 at ∼65.3 %, ALK9 at ∼60.7 %, and ALK8 at ∼57.6 %.

These results align with recent studies showing ∼10–15 % higher digestibility for DES–US systems versus alkaline controls. This enhancement is attributed to the synergistic mechanism of DES–US systems combining mild chemical solubilization with US-assisted physical disruption. Cavitation from ultrasound generates microjets and shear forces that break cell walls and loosen matrix components, facilitating solvent penetration and protein release (Kapoor et al., 2024). SEM images revealed fragmented, porous microstructures, increasing surface area for proteolytic attack.

#### 3.10.2 Solubility of extracted hazelnut proteins

Protein solubility is a critical property governing functional roles in food systems such as emulsification, foaming, and gelation. As shown in Fig. 4c, extraction method significantly influenced the solubility of hazelnut protein isolates (*p* < 0.05).

DES-extracted proteins consistently exhibited higher solubility than alkaline-derived samples. DES-GLY and DES-GLU achieved the highest solubility (∼75–76 %), while DES-SOR also remained elevated (∼70–71 %). In contrast, alkaline-extracted proteins showed significantly lower solubility, with ALK10 at ∼63 %, ALK9 at ∼61 %, and ALK8 at ∼58 %.

These results align with studies showing that DES–US systems enhance aqueous dispersibility of plant proteins by promoting partial unfolding without triggering irreversible aggregation. Comparable solubility enhancements have been documented in varying proteins, where tailored DES conditions maintained functional hydration while limiting dense reaggregation (Karimi et al., 2024; Cao et al., 2023; Dermanovic et al., 2025; Wang et al., 2025). For algal proteins, Moldes et al. (2024) similarly reported ∼72–74 % solubility with sorbitol-based DES systems, highlighting improved aqueous stability. This synergy enables the extraction of partially unfolded yet highly hydrated conformations that favor water–protein interactions and dispersion.

In contrast, alkaline extraction promotes strong pH-driven denaturation that unfolds proteins extensively but also triggers reaggregation upon neutralization and drying, yielding dense, cohesive matrices that limit water compatibility (Hanafi et al., 2024). This reduced solubility has been consistently reported in high-pH-extracted proteins (Karimi et al., 2024; Wang et al., 2025).

#### 3.10.3 Emulsifying properties of hazelnut protein

Emulsifying properties, specifically Emulsifying Activity Index (EAI) and Emulsion Stability (ES), offer critical insight into the interfacial behavior and functional versatility of plant proteins in food systems (Fig. 4d). These metrics reflect a protein's ability to adsorb rapidly at the oil–water interface and to stabilize emulsions against coalescence over time.

EAI results showed clear differences across extraction methods. DES–US-extracted proteins exhibited significantly higher EAI than alkaline-derived samples. DES-GLY achieved the highest EAI (∼15 m^2^/g), reflecting excellent interfacial adsorption, likely due to glycerol's plasticizing effect, which increases molecular flexibility and enables better unfolding and orientation at interfaces (Moldes et al., 2024). DES-SOR and DES-GLU also had elevated EAI (∼11–13 m^2^/g), consistent with partial unfolding and disruption of intramolecular interactions during extraction. In contrast, alkaline-extracted proteins showed significantly lower EAI (<6 m^2^/g), likely due to extensive pH-induced denaturation and reaggregation during neutralization and drying, which limit molecular mobility and hinder surface adsorption (Karabulut et al., 2024).

ES revealed a nuanced pattern. While DES-GLY excelled in immediate emulsification (high EAI), its ES was moderate (∼30 min). DES-SOR achieved the longest stability (∼60 min), and DES-GLU was intermediate (∼45 min). Alkaline-extracted proteins exhibited minimal ES (∼5–6 min), indicating fragile interfacial films prone to rapid coalescence. The high ES of DES-SOR, despite lower EAI than DES-GLY, suggests the formation of cohesive, viscoelastic interfacial films stabilized by hydrogen bonding and steric hindrance from residual sorbitol (Ruesgas-Ramón et al., 2017). This stabilizing effect of sorbitol-based DES systems has also been documented in cassava leaf and algal proteins (Moldes et al., 2024; Patra & Venugopal, 2025). The stabilization of viscoelastic interfacial films in DES-treated proteins can be attributed to the combined effects of hydrogen bonding and steric hindrance. Hydrogen bonds formed between protein functional groups (e.g., carbonyl, amino, and hydroxyl groups) and residual DES components promote stronger intermolecular interactions, thereby reinforcing cohesion within the adsorbed protein layer at the oil–water interface. In parallel, bulky polyol or sugar molecules, such as sorbitol and glucose, create steric hindrance that limits excessive protein aggregation and prevents film collapse. This combination of enhanced hydrogen-bonding networks and steric barriers favors the formation of a thicker, more elastic interfacial film that resists deformation, coalescence, and rupture, ultimately prolonging emulsion stability (Moldes et al., 2024). Supporting these findings, SEM images of DES-treated hazelnut proteins revealed fragmented, porous structures increasing surface-active site availability and improving interfacial packing.

#### 3.10.4 Foaming capacity and stability of hazelnut protein

**Fig. 5:**
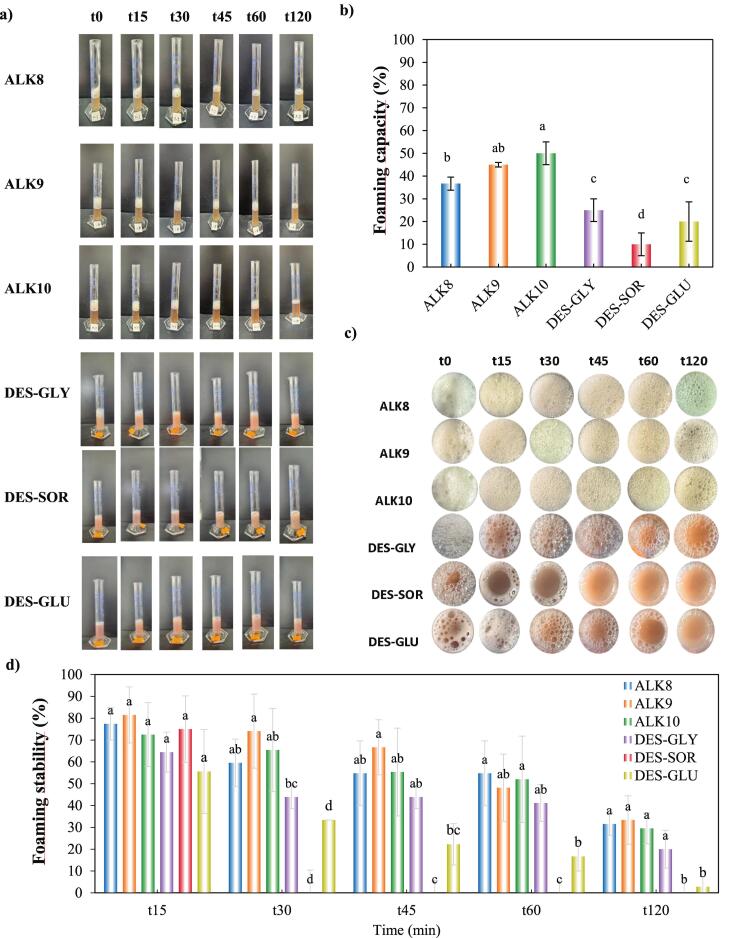
a) Images of formed foams at time stages of 0–120 min., b) Foaming capacity (%), c) Upper images of the formed foams at time stages of 0–120 min., and d) Foaming stability (%) of alkaline and DES-extracted hazelnut proteins.

The foaming properties of hazelnut protein isolates, measured as foaming capacity (FC, %) and foaming stability (FS, %), were strongly influenced by extraction method (Figs. 5a–5d). Alkaline-extracted proteins consistently demonstrated superior foaming performance. ALK10 achieved the highest FC (∼50 %), followed by ALK9 (∼45 %) and ALK8 (∼37 %) (*p* < 0.05).

This enhanced foaming under alkaline conditions is mechanistically linked to pH-induced unfolding. Elevated pH promotes deprotonation of acidic side chains, increases net negative charge, and disrupts intra- and intermolecular bonds, yielding more flexible, partially unfolded conformations. Such flexibility improves rapid adsorption at the air–water interface and alignment of hydrophobic residues to form cohesive interfacial films capable of entrapping air during whipping (Dabbour et al., 2024; Yildiz et al., 2018).

In contrast, DES-extracted proteins in this study exhibited significantly lower foaming capacities. DES-SOR and DES-GLU had especially poor FC (∼10 % and ∼ 20 %), while DES-GLY performed modestly better (∼25 %). These limitations stem from the DES environment's strong hydrogen-bonding networks and higher viscosity, which stabilize more compact, partially folded structures and restrict complete unfolding needed for interfacial activity (Anvar et al., 2025).

ALK9 and ALK10 maintained over 50 % of initial foam volume at 60 min. and ∼ 40–50 % at 120 min., suggesting cohesive, elastic interfacial films resistant to coalescence and drainage. This stability is also likely supported by higher zeta potential (strong electrostatic repulsion) and smaller, uniform particle sizes improving film integrity (Tjalsma et al., 2025). In contrast, DES-extracted proteins showed rapid foam collapse. DES-SOR and DES-GLU foams fell below 30 % stability by 45 min and under 20 % by 120 min. DES-GLY maintained relatively better stability among DES samples, suggesting glycerol's plasticizing effect promotes limited flexibility and interfacial film formation (Hewage et al., 2024). Visual assessments supported these observations: alkaline-derived foams appeared more uniform, dense, and persistent, while DES-derived foams were sparse, heterogeneous, and prone to rapid drainage and coalescence.

## 4 Conclusions

This study demonstrates that the choice of extraction strategy profoundly shapes the structural, functional, and nutritional properties of hazelnut protein isolates. Alkaline extraction remains effective for maximizing overall protein recovery and generating foaming-stable ingredients, but it induces extensive denaturation and aggregation that limit solubility and digestibility. By contrast, ultrasound-assisted DES extraction provides a milder, greener alternative capable of preserving native-like secondary structures, enhancing solubility, and improving enzymatic digestibility, while also delivering proteins with superior emulsifying properties. Importantly, these results highlight that the relative advantages of DES–US versus alkaline extraction are highly function-specific. While DES–US systems excel at producing proteins suited for emulsification and solubility-driven applications, alkaline extraction remains more suitable for foamed or aerated systems. This functional divergence emphasizes the need for tailored process design depending on the target food application. Future research should focus on further optimizing DES formulations to reduce viscosity and improve mass transfer without compromising environmental benefits. Additionally, refining post-extraction steps such as anti-solvent precipitation, membrane filtration, and drying protocols will be critical to improve the yield and recovery of functional proteins. Investigating the scalability of DES–US systems, assessing cost-effectiveness, and evaluating sensory and nutritional outcomes in finished food products will also be essential steps to translate these promising lab-scale results into viable industrial processes. Overall, valorizing hazelnut meal through such advanced extraction technologies supports broader sustainability goals by reducing agricultural waste and enabling the production of high-quality, plant-based protein ingredients for diverse food applications.

## CRediT authorship contribution statement

**Esra Kibar Balballi:** Writing – original draft, Visualization, Methodology, Investigation, Formal analysis. **Gulsah Karabulut:** Writing – review & editing, Visualization, Supervision, Project administration, Methodology, Funding acquisition, Conceptualization.

## Declaration of competing interest

The authors declare that they have no known competing financial interests or personal relationships that could have appeared to influence the work reported in this paper.

## Data Availability

Data will be made available on request.

## Appendix A Supplementary Data

Supplementary material 1 Composition and molar weight ratios of DESs. Supplementary material 1

Supplementary material 2 Multiple-reaction monitoring (MRM) conditions of target amino acids. Supplementary material 2
