# Supplementary material for: Ultrasound-assisted deep eutectic solvent extraction versus alkaline extraction: Functional and structural properties of hazelnut proteins
Source: Food Chem X. 2025 Sep 24;31:103080. doi: 10.1016/j.fochx.2025.103080 (PMC12509131; doi:10.1016/j.fochx.2025.103080)
Supplement: Supplementary material 1 — Composition and molar weight ratios of DESs. [file mmc1.docx]

**Supplementary File**

**Ultrasound-assisted deep eutectic solvent extraction versus alkaline extraction: Functional and structural properties of hazelnut proteins**

Esra Kibar Balballi^a^, Gulsah Karabulut^a,*^

^a^Department of Food Engineering, Faculty of Engineering, Sakarya University, Sakarya 54187, Türkiye

**Table S1.** Composition and molar weight ratios of DESs.

| Composition (ChCl:HBD) | pH  (25 °C) | Viscosity (mPa·s) | Refractive Index | Density (g/cm³) |
| --- | --- | --- | --- | --- |
| ChCl:Glycerol (1:2) | 5.21 ± 0.07^a^ | 56.3 ± 5.4^c^ | 1.440 ± 0.002^c^ | 1.115 ± 0.006^c^ |
| ChCl:Sorbitol (1:1) | 4.82 ± 0.05^b^ | 97.8 ± 8.6^b^ | 1.448 ± 0.003^b^ | 1.228 ± 0.009^b^ |
| ChCl:Glucose (2:1) | 4.53 ± 0.06^c^ | 181.4 ± 12.3^a^ | 1.451 ± 0.002^a^ | 1.243 ± 0.008^a^ |

*Addition with %40 distilled water. Lowercase letters indicate significant differences between different DES (*p* < 0.05) (mean ± SD, n = 3).
