# Supplementary material for: Ultrasound-assisted deep eutectic solvent extraction versus alkaline extraction: Functional and structural properties of hazelnut proteins
Source: Food Chem X. 2025 Sep 24;31:103080. doi: 10.1016/j.fochx.2025.103080 (PMC12509131; doi:10.1016/j.fochx.2025.103080)
Supplement: Supplementary material 2 — Multiple-reaction monitoring (MRM) conditions of target amino acids. [file mmc2.docx]

**Supplementary File**

**Ultrasound-assisted deep eutectic solvent extraction versus alkaline extraction: Functional and structural properties of hazelnut proteins**

Esra Kibar Balballi^a^, Gulsah Karabulut^a,*^

^a^Department of Food Engineering, Faculty of Engineering, Sakarya University, Sakarya 54187, Türkiye

**Table S2**

**Table S2.** Multiple-reaction monitoring (MRM) conditions of target amino acids.

| Amino acids | Precursor ion (m/z) | Product ion (m/z) | Fragmentor voltage (V) | Collision energy (V) |
| --- | --- | --- | --- | --- |
| Aspartic acid | 134.1 | 74.1 | 90 | 10 |
| Alanine | 90.2 | 44.2 | 80 | 4 |
| Arginine | 175.2 | 70.2 | 110 | 20 |
| Histidine | 156.1 | 110.1 | 100 | 8 |
| Cystine | 241.1 | 74.2 | 100 | 24 |
| Isoleucine | 132.2 | 69.2 | 100 | 14 |
| Glutamic acid | 148.1 | 84.2 | 80 | 12 |
| Leucine | 132.2 | 43.3 | 100 | 24 |
| Ornithine | 133.2 | 70.3 | 80 | 14 |
| Lysine | 147.1 | 84.2 | 80 | 12 |
| Glycine | 76.2 | 30.1 | 80 | 1 |
| Proline | 116.2 | 70.2 | 90 | 12 |
| Serine | 106.2 | 60.2 | 80 | 4 |
| Threonine | 120.2 | 74.2 | 80 | 4 |
| Methionine | 150.1 | 104.1 | 80 | 4 |
| Tyrosine | 182.1 | 165 | 80 | 1 |
| Phenylalanine | 166.1 | 120.1 | 80 | 6 |
| Valine | 118.2 | 72.2 | 80 | 4 |
